# A Good Story

**Published:** 1848-02

**Authors:** 


*A Good Story.*—When we were a medical student, we remember hearing Dr. R., of Boston, than whom no man can tell a story better, relate something like the following:—

"Sometime since I was called into the country to see an old lady who was afflicted more with general debility than any real disease. She requested me to go and see her old family physician, who resided near at hand, and who, she said, *understood her constitution.* I accordingly called upon the old doctor, who had then ceased his active practice, made known my errand, and received an account of the old lady's constitution. As I was about leaving, the old gentleman, looking me full in the face, said, 'Doctor, did you know that I was a great surgeon in my day? 'No, sir.' I replied, 'I did not.' 'Well, I was,' continued he. 'A number of years ago, I was called into B., to consult with the present eminent Dr. W. about taking off the leg of a black man. I arrived fifteen minutes past the time appointed, and Dr. W. was just taking up the knife to commence the operation of amputating tho negro's leg. I asked him what he sent for me for? He said, to give my opinion as to taking off this man's leg. 'Well, why did you not wait, then, till I came?' 'Because, sir, we thought something had detained you, and you wonld not come.'

"After examining the leg, I was well satisfied it could be saved. I took him into my chaise; brought him up to a black woman's house near by, and cured his leg. He has now been my porter for twenty years, and carried bundles and loads upon that leg ever since. This is the way *I was a great surgeon—not by cutting off legs but by saving them.*"

MORAL.—Many legs might have been saved, that have been cut off.—*Journal of Health.*

